# Decentralized environmental health governance in a mining municipality: An interpretive analysis from Obuasi, Ghana

**DOI:** 10.1016/j.puhip.2026.100788

**Published:** 2026-04-03

**Authors:** Charles Owusu-Aduomi Botchwey, Albert Ahenkan, Lawrencia Aggrey-Bluwey, Susuana Adutwumwaa, Doreen Oparebea Odei, Emmanuel Sei Nkpetri

**Affiliations:** a University of Education, Winneba, Faculty of Health, Allied Sciences and Home Economics Education, Department of Health Administration and Education, Post Office Box 25, Winneba, Central Region, Ghana; b University of Ghana, School of Business, Department of Public Administration and Centre for Climate Change and Sustainable Studies, Ghana; c University of Education, Winneba∗, Faculty of Health, Allied Sciences and Home Economics Education, Department of Health Administration and Education, Post Office Box 25, Winneba, Central Region, Ghana; d University of Cape Coast, Faculty of Social Sciences Education, Department of Business and Social Sciences Education, University Post Office, Cape Coast, Central Region, Ghana; e Presbyterian Women's College of Education, Aburi, Department of Social Studies, Post Office Box 19, Aburi, Eastern Region, Ghana

**Keywords:** Decentralization, Environmental health, Mining communities, Local governance, Ghana

## Abstract

**Objectives:**

Decentralization is widely promoted as a strategy for strengthening local governance and improving environmental health outcomes, particularly in settings exposed to pollution and unsafe living conditions. However, the extent to which decentralization delivers these benefits in mining-dependent municipalities remains unclear. This study examined how decentralized governance shapes environmental health management in the Obuasi Municipality of Ghana, with particular attention to institutional authority, capacity constraints, and community participation.

**Study design:**

This study employed a qualitative interpretivist design.

**Methods:**

Semi-structured interviews were conducted with thirty stakeholders, including municipal officials, environmental health workers, community leaders, and residents in mining-affected communities. Data were analyzed using reflexive thematic analysis to identify patterns related to governance structures, capacity, participation, and environmental health risks.

**Results:**

Four main themes emerged from the analysis. These are authority gaps within decentralized governance structures, institutional capacity constraints affecting preventive health functions, reactive community participation driven by health crises, and governance pressures created by mining-related environmental risks. Participants described how local authorities are expected to manage environmental health outcomes despite lacking regulatory authority, financial resources and technical capacity to address mining-related hazards.

**Conclusions:**

Environmental health outcomes in mining municipalities cannot be improved through administrative decentralization alone. Strengthening environmental health governance requires clearer regulatory authority at the local level, targeted investments in technical and financial capacity, and more inclusive, health-focused engagement mechanisms to protect community health in high-risk environments.

## 1 Introduction

Decentralized governance has been central to Ghana's public sector reforms for more than three decades, with the aim of improving responsiveness, strengthening local accountability and enhancing the capacity of Metropolitan, Municipal and District Assemblies (MMDAs) to deliver public services [1]. Although these reforms were initially framed around local development and service delivery, they have increasingly intersected with the broader challenge of protecting the environmental determinants of health such as clean water, safe air, sanitation, waste management and land-use regulation, which are now recognized as core components of public health systems [WHO, 2]. In this sense, environmental governance is a fundamental health policy concern.

In Ghana's mining municipalities, this connection is particularly prominent. Obuasi, located in the Ashanti Region, is one of the country's most well-known mining zones, with more than a century of industrial gold mining and a rapidly expanding artisanal mining economy [[3], [4], [5]]. These activities have created complex environmental exposures such as chemical contamination of water bodies, particulate air pollution, soil degradation, deforestation, abandoned mining pits, and sanitation breakdowns, that directly influence disease patterns and public health outcomes at the community level [6]. Traditional environmental challenges such as open defecation, uncollected waste and poor drainage have become intertwined with mining-related hazards, creating a landscape where environmental policy failures translate into increased health risks [7].

Environmental health concerns in mining areas are also reflected in broader public health trends. According to Ghana Health Service reports [8], malaria remains one of the leading causes of outpatient morbidity in mining regions of the Ashanti Region, while waterborne diseases such as diarrheal infections continue to contribute substantially to the burden of disease in communities with compromised water and sanitation systems. These patterns reinforce the close relationship between environmental management, local governance capacity and preventive public health outcomes in mining municipalities.

Despite the severity of these issues, environmental health governance in mining areas operates within a fragmented institutional architecture. National regulatory agencies including the Environmental Protection Agency (EPA), the Minerals Commission and the Water Resources Commission exercise significant authority over mining concessions, environmental permitting and impact monitoring [9]. Meanwhile, MMDAs are responsible for the environmental health responsibilities closest to communities. These include waste management, environmental sanitation, food safety inspections, enforcement of local by-laws, and the work of environmental health officers. These tasks are central to the prevention of communicable diseases and the safeguarding of environmental conditions that shape non-communicable disease risks [10]. However, local governments often struggle to fulfil these responsibilities due to limited funding, shortages of environmental health personnel, inadequate logistics and weak enforcement mechanisms [11]. For example, one of the municipality's core responsibilities is to ensure access to safe drinking water, yet polluted rivers and artisanal mining undermine water safety, placing additional burdens on the Ghana Health Service, municipal health facilities and community disease surveillance systems. The environmental degradation resulting from mining activities therefore intensifies municipal health responsibilities, increases the need for preventive interventions and contributes to the spread of waterborne diseases, respiratory illnesses and injuries [3,12].

This situation highlights a fundamental problem. Although decentralization assigns environmental and preventive health responsibilities to local governments, municipalities often lack the authority and institutional capacity required to regulate the underlying drivers of environmental degradation. [13]. Mining companies operate with substantial bargaining power, and artisanal mining is reinforced by economic necessity, weak enforcement and political sensitivities [4]. As a result, environmental governance becomes a health policy challenge that local governments can neither ignore nor fully control.

This study is situated within these tensions. It examines decentralized environmental governance in the Obuasi Municipality with particular attention to how governance failures and institutional constraints translate into public health risks. It argues that the environmental impacts of mining are inseparable from the health consequences experienced by local populations, and therefore, decentralized environmental governance should be understood as a component of the health system. The study pursues four objectives. To examine how decentralization functions as a governance tool for environmental sustainability and environmental health protection, to explore capacity challenges shaping municipal efforts to manage environmental health risks, to assess the nature of community participation in environmental and health-related decision-making and to analyze lessons emerging from mining-related environmental pressures that affect local health systems and public health outcomes. This study contributes to a growing body of scholarship recognizing that environmental conditions are core components of local health systems. It offers evidence of how decentralization reforms, originally intended to strengthen local governance, can only support public health when governance structures, institutional capacity and community engagement are effectively aligned.

### 1.1 Conceptual framing

The analysis for this study draws on three interrelated conceptual perspectives that together provide a coherent lens for examining decentralized environmental governance as a form of public health governance. These are polycentric governance, institutional capacity theory and participatory environmental health management. Environmental governance is inherently health governance because environmental exposures are among the most powerful determinants of population health. As global health governance literature emphasizes, protecting water, air, land and sanitation systems is central to preventing disease and sustaining community health [14]. The WHO similarly frames environmental management as a core public health function, arguing that health systems cannot achieve population-level protection without coordinated environmental regulation and preventive governance structures [2].

Polycentric governance theory proposes that effective management of complex societal challenges, such as environmental degradation and public health protection, requires multiple centers of authority interacting across different scales [15]. In Ghana, environmental health governance is distributed across national regulators, municipal authorities, traditional councils, mining companies and community organizations [16,17]. This plurality offers the potential for responsiveness and local knowledge integration, but also creates fragmentation, mandate overlaps and blurred accountability. When national agencies issue mining permits without local consultation, or when municipalities lack the authority to enforce by-laws against powerful actors, polycentric arrangements can amplify governance failure.

Institutional capacity theory complements this by emphasizing the resources necessary for decentralized governance units to perform effectively. Local governments require financial resources, skilled environmental health personnel, monitoring technologies, enforcement support and stable administrative systems to fulfil their assigned roles [11]. In mining municipalities, these capacities must include the ability to assess environmental health risks, manage sanitation systems, coordinate disease prevention activities and conduct health education. Where these capacities are weak, as commonly found in resource-constrained municipalities, the preventive health functions tied to environmental management become compromised.

The third framing, participatory environmental health management, highlights the importance of community involvement in identifying environmental health risks, shaping local interventions and monitoring compliance [18]. Community participation is a core principle of Ghana's decentralization system, yet research shows that in mining areas, participation often becomes symbolic or reactive, emerging only after environmental damage leads to visible health consequences [19]. Participation is influenced by trust in authorities, perceived fairness in decision-making and the socioeconomic reliance on mining. These dynamics shape whether environmental health risks are prevented early or allowed to escalate.

**Fig. 1:**
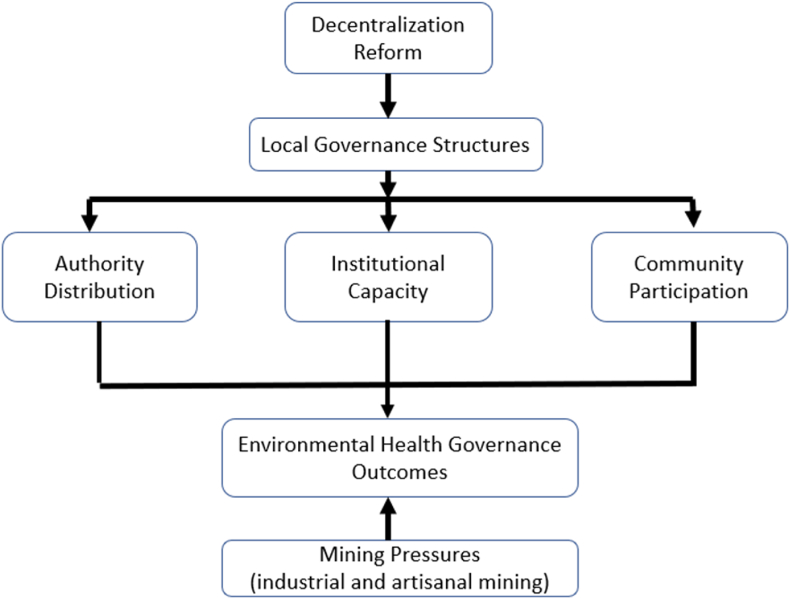
Analytical framework for decentralized environmental health governance in mining municipalities.

**Source**: Authors' conceptualization based on the study's conceptual framing

These three perspectives provide an integrated analytical framework for interpreting environmental health governance in mining municipalities. Polycentric governance explains the distribution of authority across multiple institutional actors and the coordination challenges that arise from overlapping mandates [15,16]. Institutional capacity theory highlights the material and technical resources required for decentralized authorities to perform their responsibilities effectively [13,20]. Participatory environmental health management emphasizes the role of community engagement and local legitimacy in shaping preventive health responses [21]. In combination, these lenses allow the study to examine how authority structures, institutional capacities and community participation interact to influence environmental health outcomes under decentralization. This analytical framework is presented in Fig. 1 below.

### 1.2 Analytical logic of the framework

The analytical logic of the framework is grounded in the assumption that decentralization shapes environmental health governance through the institutional arrangements it creates at the local level. Decentralization reforms establish municipal governance structures that are responsible for environmental management and preventive public health functions. The effectiveness of these structures depends on three interrelated institutional dimensions. These are the distribution of regulatory authority across governance levels, the institutional capacity available to local authorities, and the nature of community participation in environmental decision-making. These governance dynamics operate within a broader context of mining pressures, including industrial and artisanal mining activities that generate environmental exposures and intensify public health risks. The framework therefore illustrates how decentralization interacts with authority structures, institutional capacity and community engagement to influence environmental health governance outcomes in mining municipalities. Because this study adopts a qualitative interpretivist approach, the relationships represented in the framework are not intended to be statistically tested or modeled quantitatively. Instead, the framework serves as an analytical guide that structures the interpretation of stakeholder experiences and helps explain how governance dynamics shape environmental health conditions in the study setting.

## 2 Methods

### 2.1 Study design

A qualitative interpretivist design was adopted because the study sought to understand how decentralization, environmental governance and health policy responsibilities are experienced and interpreted by municipal officials, community stakeholders and residents. Interpretivist inquiry is appropriate for unpacking lived experiences, perceptions of authority, institutional tensions and social meanings attributed to environmental health risks [21].

### 2.2 Research setting

The study was conducted in the Obuasi Municipality, a mining-dependent district characterized by significant environmental health challenges including water pollution, air quality deterioration, poor sanitation, and increased vector breeding sites [22]. These conditions make Obuasi an appropriate setting for exploring how decentralized environmental governance affects public health outcomes.

### 2.3 Sampling strategy

Purposive sampling was used to recruit participants with direct involvement in environmental governance and environmental health such as municipal officers, environmental health inspectors, community leaders, unit committee members, traditional leaders, residents in mining-affected communities and officers from national agencies present at the municipal level [23]. A total of 30 participants were selected after saturation was reached when additional interviews no longer produced new themes and insights concerning decentralization, environmental governance, and environmental health risks. The characteristics of the selected participants are presented in Table 1 below.

**Table 1:** Participant characteristics.

Participant Category	Number	Gender (M/F)	Institutional Affiliation	Average Years of Experience
Municipal officials	7	5M/2F	Municipal Assembly	9 years
Environmental health officers	6	4M/2F	Environmental Health Unit	8 years
Community leaders	5	4M/1F	Traditional/Community leadership	11 years
Unit committee members	4	3M/1F	Local governance structures	7 years
National agency officers	3	2M/1F	EPA/related agencies	10 years
Community residents	5	3M/2F	Mining affected communities	-

Representatives of mining companies were not included in the participant sample. Access to corporate officials was limited during the data collection period, and the study focused primarily on governance experiences at the municipal and community levels where environmental health impacts are most directly experienced. While the perspectives of mining companies would provide an important complementary dimension to the analysis, the absence of these actors reflects the practical challenges of accessing corporate stakeholders in governance research [3]. This limitation is acknowledged in the discussion of study limitations.

### 2.4 Data collection

Semi-structured, in-depth interviews were conducted using an interview guide that explored decentralization, environmental health risks, institutional authority, coordination challenges, mining impacts and community participation. Participants were also invited to reflect on possible policy responses and institutional reforms that could strengthen environmental and public health governance in mining communities, which informed the development of the policy recommendations discussed later in the paper.

Interviews were conducted between 1st March, 2024 to 29th May, 2024 and lasted 30 to 60 min, were conducted in English Language, audio-recorded with consent and transcribed verbatim. Field notes captured non-verbal observations and researcher reflexivity.

### 2.5 Ethical considerations

Ethical approval was obtained from the University of Ghana School of Business with reference number: UGBS/COAB/20.05.07. Participants were briefed on confidentiality, anonymity, voluntary participation and the right to withdraw. All identifiers were removed from transcripts and audio files were securely stored.

### 2.6 Data analysis

Data were analyzed using reflexive thematic analysis following the six-stage process outlined by Braun and Clarke [24]. The analysis began with repeated reading of the transcripts to achieve familiarization with the data. Initial codes were then generated inductively from participants’ narratives while also drawing on deductive categories derived from the conceptual framing, particularly authority, institutional capacity, participation and perceived health risks. Coding was conducted manually by the lead researcher and subsequently reviewed by another member of the research team to enhance analytical rigor. Differences in interpretation were discussed and resolved through iterative comparison of coded extracts and analytic memos.

Reflexive memoing [25,26] was used throughout the analysis to document emerging interpretations, contextual reflections and possible researcher assumptions. Saturation was considered to have been reached at the level of meaning when additional interviews no longer produced new conceptual insights related to decentralization, governance capacity or environmental health experiences. Themes were refined through constant comparison across participant groups, including municipal officials, environmental health officers and community residents, to ensure that the analysis captured diverse perspectives on governance challenges.

### 2.7 Ensuring trustworthiness

Credibility was strengthened through triangulation across participant groups, including municipal officials, environmental health officers and community residents. For example, accounts of enforcement challenges reported by municipal officers were compared with community experiences of continued exposure to waste and polluted water sources to identify convergence and divergence in perspectives. In practice, this involved comparing how municipal officials described enforcement constraints with how community residents experienced continued exposure to waste, polluted water and vector breeding conditions, allowing the analysis to identify both agreement and points of tension between institutional and lived perspectives. Prolonged engagement with the data through repeated reading of transcripts also supported deeper interpretation of participants’ meanings.

Transferability was also supported through detailed description of the study setting, including the governance structure of the Obuasi Municipality and the environmental health conditions associated with mining activities, allowing readers to assess the applicability of findings to similar contexts. Further, dependability was ensured through the maintenance of an audit trail documenting key stages of the research process, including interview procedures, coding decisions, theme development and revisions made during analysis. The audit trail included dated records of coding frameworks, revisions to theme definitions, and documented decisions on how conflicting interpretations were resolved during analysis. Analytic memos were also used to track how initial codes evolved into broader themes over time.

Lastly, confirmability was enhanced through reflexive journaling, which documented the researchers' assumptions, decisions and potential influences on interpretation. Regular discussions among the research team were used to critically examine emerging interpretations and ensure that findings remained grounded in participants’ accounts.

### 2.8 Researcher positionality

Because research on governance and environmental regulation can involve politically sensitive issues, the research team reflected carefully on their positionality throughout the study. The researchers are affiliated with academic institutions in Ghana and approached the study from a public health and governance perspective rather than from regulatory or corporate institutions involved in mining activities. While this positioning facilitated access to municipal officials and community members, it also required awareness of potential power dynamics during interviews, particularly when discussing institutional weaknesses or enforcement challenges.

Participants were informed that the research was independent of regulatory agencies and that their responses would remain confidential. Reflexive memoing [25,26] was further used to document how the researchers’ institutional positions, professional backgrounds and assumptions about decentralization might influence interpretation. During analysis, particular attention was paid to differences between the perspectives of municipal officials and those of community residents in order to preserve the interpretive nature of the inquiry.

## 3 Results

This section presents the findings of the study, organized around the four research objectives. The analysis followed Braun and Clarke's reflexive thematic analysis approach. Direct quotations are used to illustrate participants' perspectives, followed by brief analytical interpretations that clarify the meaning and significance of the accounts.

### 3.1 Objective 1: Decentralization as a governance tool for environmental sustainability and environmental health

Analysis of participants’ accounts under this objective generated two interrelated themes. The first theme captures how decentralization has created visible local governance structures for environmental and public health management without transferring corresponding regulatory authority. The second theme highlights how fragmented and overlapping institutional mandates undermine coordinated environmental health governance at the municipal level.

#### 3.1.1 Theme 1: Decentralization creates visible local governance structures but not meaningful environmental health authority

Participants consistently described decentralization as creating formal platforms for environmental and public health oversight, including the Environmental Health Unit, the Municipal Planning Committee and community complaint channels. These structures place the Municipal Assembly at the center of preventive health functions such as sanitation, safe water practices, disease prevention campaigns and waste regulation. However, participants emphasized that meaningful authority to regulate mining impacts, control environmental hazards, or sanction environmentally harmful operations remained firmly controlled by national agencies. This authority gap undermines the municipality's ability to protect environmental health.

A senior municipal officer explained: *“People report environmental health problems to us, polluted water, dust, refuse, but when it comes to major mining issues, we don't have the mandate to act. We send reports upward and wait”* - Official 7. This reflects more than a simple imbalance of power. It reveals a governance arrangement in which responsibility is devolved in administrative terms, while decision-making authority over high-impact environmental issues remains centralized. This separation constrains the ability of municipal actors to act on locally observed health risks, forcing them into a reporting role rather than a regulatory one.

A community health volunteer similarly stated: *“When we complain about water contamination, the Assembly tells us they are waiting for EPA or Minerals Commission. But the water is still what our children drink every day”* - Community Health Rep 1.

Another participant expressed frustration with the governance disconnect: *“Decentralization puts the environmental health burden on us, but the power to prevent the problems sits in Accra”* - Environmental Health Officer 3.

Although decentralization enhances visibility of municipal responsibilities, the lack of substantive decision-making power weakens its effectiveness as a health governance mechanism. Taken together, these accounts suggest that decentralization reconfigures the location of accountability without redistributing the authority needed to act on environmental health threats. As a result, municipal institutions become the first point of contact for community complaints but remain structurally limited in their ability to resolve them, reinforcing a cycle of expectation without response. This disconnect leaves local governments accountable for health impacts they cannot control.

#### 3.1.2 Theme 2: Fragmented mandates undermine coherent environmental health protection

Participants described how overlapping mandates among the EPA, Water Resources Commission, Minerals Commission, Municipal Assembly and traditional authorities create confusion in environmental health decision-making. This fragmentation contributes to delays in addressing health risks arising from mining activities, waste mismanagement and unsafe water sources.

A planning officer summarized the challenge: *“EPA will say one thing, Minerals Commission will say another, and we at the Assembly have to explain these contradictions to community members who just want clean water and clean air”* - Planner 2.

A community resident added: *“When you chase one agency, they say it is another agency's job. Meanwhile, we are the ones suffering from the bad water and the dust”* - Resident 4.

Another officer pointed out the health implications directly: *“Because responsibilities are scattered, health problems escalate before any agency responds. By the time action comes, the community is already dealing with diarrhea cases and respiratory complaints”* - Health Officer 2.

These institutional overlaps point to a coordination problem within polycentric governance arrangements, where multiple actors operate with overlapping mandates but without clear mechanisms for joint decision-making. This fragmentation shifts the burden of navigating institutional complexity onto communities, while simultaneously delaying responses to environmental health threats.

### 3.2 Objective 2: Capacity challenges affecting environmental and public health governance

For the second objective, three key themes emerged in relation to capacity constraints under decentralized environmental and public health governance. These themes relate to chronic financial limitations affecting preventive health services, inadequate technical expertise for managing environmental health risks, and weak enforcement capacity that exposes communities to avoidable health hazards.

#### 3.2.1 Theme 1: Chronic funding delays and under investment weakens preventive health systems

Participants repeatedly noted that the District Assembly Common Fund, which should support environmental health activities, is chronically delayed and often inadequate. The lack of predictable financing undermines basic preventive health services such as refuse collection, drain clearing, larval control interventions, sanitation inspection and health education campaigns.

A municipal finance officer stated: *“We are supposed to* fund *environmental health and sanitation from the Common* *Fund, but when it delays, everything delays, including disease prevention”* - Finance Officer 1.

A sanitation supervisor highlighted the operational struggles: *“We want to do regular inspections and health sensitization, but without fuel, logistics, or basic tools, environmental health work becomes reactive instead of preventive”* - Sanitation Officer 3.

A mother in a mining-affected community explained the consequences: *“When the drains are not cleaned because the Assembly has no money, mosquitoes take over. Then our children fall sick with malaria”* - Resident 9.

These accounts suggest that financial constraints restructure how environmental health governance is practiced at the municipal level. Rather than enabling routine preventive action, limited and unpredictable funding pushes institutions toward short-term, reactive responses, which allows environmental risks to accumulate before intervention occurs. Preventive activities are deprioritized, and responses become reactive, which increases the likelihood that environmental conditions deteriorate before action is taken.

#### 3.2.2 Theme 2: Limited technical expertise restricts the Municipality's ability to address environmental health risks

Participants emphasized the lack of specialized environmental health skills needed to interpret mining-related exposures, assess water safety, conduct risk mapping, or engage mining companies from a technical standpoint. This weakens the municipality's power in environmental health negotiations and limits evidence-based decision-making.

A technical officer explained: *“Mining companies have environmental scientists, engineers and health and safety professionals. We don't have equivalent expertise at the Assembly level”* - Technical Officer 5.

A community leader also noted: *“We report concerns about dust, bad water, or noise, but the Assembly doesn't have the experts to test or measure anything, so the complaints feel like they go nowhere”* - Community Leader 7.

Another environmental health officer added: *“Without the right technical skills, we can't fully assess how mining affects air quality or water quality. That makes it difficult to take action or communicate health risks to the public”* - Environmental Health Officer 4.

These responses indicate that limited technical capacity not only weakens evidence-based decision-making but also undermines the municipality's ability to engage credibly with more technically equipped actors such as mining companies and national regulators. This imbalance reduces the influence of local authorities in environmental decision-making processes that directly affect community health.

#### 3.2.3 Theme 3: Weak enforcement creates health vulnerabilities in mining communities

Enforcement challenges were repeatedly linked to increased health risks. Participants described insufficient environmental health staff, lack of transport, absence of police support and political pressures as major barriers to enforcing sanitation by-laws, food hygiene regulations, mining restrictions and community safety rules.

An enforcement officer stated: *“We have the laws, but we don't have the personnel or vehicles to enforce them. So illegal mining, unsafe disposal and pollution continue, and these affect public health directly”* - Enforcement Officer 2.

A community participant added: *“People dump waste in the open because they know nobody will arrest them. When it rains, all that waste enters the streams. Then we drink that water”* - Resident 6.

Another municipal official explained: *“Environmental health inspectors cannot confront offenders who have political backing. This limits our ability to protect community health”* - Official 8.

These accounts point to a broader institutional constraint in which enforcement is shaped not only by logistical limitations but also by political and social dynamics. The inability to sanction offenders, particularly those with economic or political influence, reflects how enforcement practices are negotiated rather than consistently applied, weakening the protective function of environmental health regulations.

### 3.3 Objective 3: Community participation in environmental and health governance

For objective 3, the data revealed two main themes concerning community participation in environmental and health governance. The first theme reflects the largely reactive nature of community engagement, which is often triggered by emerging health crises rather than sustained institutional participation. The second theme highlights how mistrust between communities and authorities undermines collaborative environmental health action.

#### 3.3.1 Theme 1: Participation is informal, irregular and triggered mostly by health crises

Although decentralization emphasizes community engagement, participants described community involvement as mostly reactive. Communities tend to mobilize when environmental conditions begin affecting health, such as when water becomes unsafe, mosquitoes increase, or polluted air worsens respiratory issues. Participation becomes crisis-driven because communities often perceive environmental governance processes as distant or unresponsive, engaging only when deteriorating conditions begin to threaten health and daily survival, such as unsafe water, surging malaria cases, or visible pollution.

A community member summarized this dynamic: *“People come out only when things get really bad, when children start getting sick or the river turns brown”* - Resident 3.

A unit committee member noted: *“We try to involve communities in planning, but attendance is low unless the issue is already affecting their health”* - Unit Committee Member 2.

A municipal health officer explained: *“Environmental health problems drive participation more than environmental governance structures do. People respond to disease risks”* - Health Officer 1.

This suggests that participation in environmental governance is structured less by formal institutional processes and more by immediate health concerns. Communities engage when environmental conditions begin to threaten health and livelihoods, indicating that participation is reactive and risk-driven rather than embedded within routine governance structures.

#### 3.3.2 Theme 2: Mistrust and perceived exclusion undermine collaborative environmental health action

Participants frequently mentioned distrust among communities, the Assembly and national agencies. Residents feel excluded from decisions involving mining permits, resource allocation and environmental enforcement, particularly when these decisions influence community health.

A community leader stated: *“People feel the Assembly listens more to mining companies than to the community. That is why there is mistrust”* - Community Leader 4.

A resident added: *“We don't get information about mining or environmental health risks early enough. By the time we know, the damage is already done”* - Resident 8.

A municipal official acknowledged the communication gaps: *“Sometimes we ourselves are not informed by national agencies. That makes it hard to build trust with the community”* - Official 5.

These accounts suggest that mistrust operates as a barrier to effective governance by limiting information flow, reducing community willingness to engage with authorities, and weakening compliance with environmental health interventions. This undermines the collaborative relationships required for sustained environmental health management.

### 3.4 Objective 4: Lessons from environmental pressures in mining communities for health systems and policy

Two themes were identified in relation to lessons emerging from environmental pressures in mining communities. These themes capture how mining activities intensify environmental health risks beyond the response capacity of decentralized institutions, and how economic dependence on mining creates difficult trade-offs between livelihood needs and long-term health protection.

#### 3.4.1 Theme 1: Mining exposures intensify public health risks and reveal governance gaps

Participants widely described mining as a powerful driver of environmental conditions that worsen public health: unsafe water, air pollution, stagnant water pits, food contamination, noise and community safety hazards.

A resident explained: *“The river we used to drink from is now polluted. People still rely on it when their taps stop flowing. That is why we see more diarrhea cases”* - Resident 10.

An environmental health officer stated: *“The abandoned pits are breeding grounds for mosquitoes. Malaria becomes worse in those communities”* - Environmental Health Officer 2.

A municipal officer added: *“Mining intensifies environmental health problems faster than we can respond, and our capacity is too limited to keep up”* - Official 9.

These accounts indicate that participants understand mining as a central driver of environmental conditions that heighten perceived health risks. This reflects a governance gap in which the scale and intensity of environmental change generated by mining outpaces the capacity of decentralized institutions to monitor, regulate, or mitigate its public health implications.

#### 3.4.2 Theme 2: Economic dependence on mining generates Health–Environment trade-offs

Participants described how communities rely heavily on mining income, even while acknowledging its health risks. This creates complex trade-offs between livelihoods and preventive health.

A resident explained: *“We know the mining affects our health, but many families depend on it to survive. It's hard to choose between food and clean water”* - Resident 5.

A municipal official added: *“When people are struggling economically, enforcing environmental health by-laws becomes politically sensitive”* - Official 3.

A community leader reflected: *“The health risks are real, but people need jobs. This tension affects participation and enforcement”* - Community Leader 3.

This highlights how economic dependence on mining reshapes governance priorities by creating trade-offs between livelihood security and long-term health protection. In this context, enforcement of environmental health regulations becomes politically sensitive, as regulatory actions may threaten local income sources, thereby constraining institutional responses.

## 4 Discussion

This study set out to examine decentralized environmental governance in a mining-dependent municipality, with particular attention to how the structures, capacities and experiences of local governance actors intersect with environmental health outcomes. The findings reveal a governance landscape where decentralization provides proximity and administrative visibility but lacks the authority, resources and coherence required to protect environmental determinants of health. A central finding is that while decentralization has created local structures for environmental and public health management, these structures remain constrained in their ability to regulate key environmental drivers, reinforcing patterns observed in the findings. This finding aligns with earlier critiques of Ghana's decentralization reforms, which note that authority over key environmental decisions remains centralized despite rhetorical commitment to devolution [1,27]. Polycentric governance theory acknowledges the potential for multi-level systems to enhance responsiveness but warns that such systems can produce fragmentation when authority is not clearly aligned across nodes [15]. The experiences of municipal officers in Obuasi demonstrate precisely this problem. This is consistent with studies in other African mining jurisdictions, where local authorities remain peripheral to regulatory processes dominated by central agencies and private companies [4]. However, the present study diverges from literature that suggests decentralization naturally strengthens local legitimacy [28,29]. In Obuasi, decentralization increases expectations without increasing power, thereby undermining public trust.

The capacity challenges identified in this study, insufficient funding, shortages of technical expertise and weak enforcement mechanisms, reinforce well-established observations that the success of decentralization depends heavily on the institutional capacity of local governments [11]. Municipal officials described how environmental health functions, including sanitation enforcement, water safety monitoring, malaria prevention and food hygiene inspections, are routinely compromised by delayed funding and inadequate logistics. This aligns with [22], who document similar constraints in mining communities. However, the present findings go further by showing how these capacity limitations create specific vulnerabilities for public health, particularly in relation to waterborne diseases, respiratory illnesses and vector-borne infections.

The study also shows that community participation in environmental and health governance remains inconsistent, reactive and influenced by mistrust. This resonates with participatory governance scholarship, which highlights that participation often becomes symbolic when information is unevenly distributed and when communities lack meaningful influence over decisions [19]. What distinguishes the present findings is the observation that community participation intensifies only when environmental degradation begins producing visible health effects. This suggests that, in mining municipalities, environmental governance is experienced primarily through health rather than administrative or regulatory lenses. This nuance extends the literature by illustrating how environmental health risks, rather than environmental concerns alone, serve as the primary triggers for community mobilization.

These findings also resonate with broader debates on environmental justice and political economy in resource extraction settings [30,31]. Mining activities generate economic benefits while simultaneously redistributing environmental health risks to local communities [4,31]. In such contexts, governance decisions are shaped not only by institutional arrangements but also by power relations between national regulators, private companies and local authorities. The reluctance to enforce environmental regulations described by participants may therefore reflect political and economic considerations that extend beyond local administrative capacity.

Another important dimension emerging from the findings concerns the role of traditional authorities within the decentralized governance landscape. Although formal regulatory authority lies with municipal assemblies and national environmental agencies, traditional leaders remain influential actors in land allocation, dispute mediation and community mobilization [32]. Participants indicated that traditional authorities often serve as intermediaries between communities and formal institutions, particularly when environmental concerns escalate into health crises. This illustrates the hybrid nature of governance in mining municipalities, where statutory decentralization mechanisms coexist with customary authority structures. Understanding how these governance systems interact is essential for designing environmental health interventions that are both institutionally legitimate and locally responsive.

The study also makes a distinct empirical and conceptual contribution to the literature on decentralization and environmental governance. While previous studies have documented authority-capacity gaps in decentralized systems [13,33], this study demonstrates how these gaps manifest specifically in mining-dependent municipalities where environmental exposures translate directly into public health risks. The findings show that decentralization creates administrative proximity between governance institutions and affected communities, yet regulatory authority remains concentrated at higher levels of government. By documenting how this contradiction operates within a mining municipality, the study contributes new empirical insight into the governance dynamics linking decentralization, environmental risk and public health. The study further reveals how mining pressures expose deeper governance weaknesses and generate health-environment trade-offs. Residents described balancing livelihood needs against long-term health risks, a tension that has been documented in artisanal mining research [4]. However, the findings here highlight how these economic decisions ripple through the municipal health system by increasing demand for preventive and curative health services. This demonstrates the interconnection among economic precarity, environmental degradation and health system burdens.

In all, the findings from this current study suggest that authority, institutional capacity, community participation and mining-related environmental pressures interact to produce system-level governance vulnerabilities. Limited regulatory authority constrains the ability of municipal institutions to address environmental risks. At the same time, resource shortages and technical capacity gaps weaken the effectiveness of existing governance structures. These constraints are further complicated by community mistrust and economic dependence on mining, which shape participation patterns and enforcement dynamics. Rather than operating as isolated challenges, these factors reinforce one another, producing a governance environment in which environmental health risks accumulate faster than institutions can respond.

### 4.1 Conclusion, policy implications, and recommendations

This study examined decentralized environmental governance in the Obuasi Municipality to understand how authority, capacity and participation shape environmental health conditions in a mining-dependent context. The findings demonstrate that decentralization provides a structural foundation for local involvement in environmental and health governance but fails to equip municipal assemblies with the authority and resources required to fulfil their preventive health responsibilities. Conceptually, the study highlights a fundamental contradiction within decentralized environmental governance systems in extraction-dependent settings. Administrative responsibility is located close to affected communities, while regulatory authority remains institutionally distant.

The environmental exposures associated with mining including unsafe water, airborne particulate matter, stagnant water pits and sanitation challenges, create significant public health risks that local governments are expected to address without adequate decision-making power or technical capacity. Community participation remains inconsistent and is often driven by acute health concerns rather than institutionalized engagement processes. As a result, the municipality is positioned at the intersection of environmental degradation and public health vulnerability, responsible for outcomes it has limited control over.

A particularly important contribution of this study is the identification of community participation as health-driven rather than policy-driven. Rather than emerging primarily through formal governance mechanisms, participation often occurs only when environmental degradation produces visible health threats. This finding highlights the central role of perceived health risk in mobilizing community engagement in environmental governance.

The findings carry specific implications for health policy and management in mining municipalities such as Obuasi. First, decentralization frameworks should include ring-fenced environmental health funds within the Obuasi Municipal Assembly's budget, earmarked for routine sanitation activities, water quality monitoring of streams affected by mining, and vector control interventions in communities with abandoned mining pits. Next, joint environmental monitoring platforms should be established at the Obuasi Municipal Assembly level, with scheduled monthly coordination meetings involving the EPA, Minerals Commission and Environmental Health Unit to review water quality data, sanitation reports and community complaints from mining-affected areas These platforms should meet regularly to review mining-related environmental data and coordinate responses to emerging health risks.

Thirdly, mining companies operating within the municipality should be required to contribute to locally administered environmental health surveillance systems, including periodic water testing of community sources and support for municipal health outreach activities in high-risk communities. Fourth, integration between the Municipal Environmental Health Unit and the Ghana Health Service in Obuasi should be strengthened so that environmental risk indicators such as water contamination and waste accumulation are incorporated into routine disease surveillance and early warning systems.

Finally, communication protocols between national regulatory agencies and the Municipal Assembly should be formalized to ensure that local authorities receive timely information on mining permits, environmental assessments and compliance reports, enabling more proactive environmental health management.

### 4.2 Limitations and suggestions for future research

This study involved 30 participants, which is appropriate for qualitative depth but does not represent all stakeholder groups involved in environmental or health governance. The findings are specific to Obuasi and may not fully apply to municipalities with different types of environmental pressures. As a qualitative study, the analysis relies on self-reported experiences and perceptions, which may be influenced by personal expectations or political dynamics. Additionally, the study did not include direct environmental or epidemiological measurements, which could have strengthened the assessment of environmental health risks.

Future studies should explore comparative analyses across multiple mining municipalities to identify common environmental health governance challenges and context-specific differences. Integrating environmental health monitoring, such as water quality testing, air quality measurements and malaria vector surveillance, would enhance understanding of the health implications of governance shortcomings. Quantitative assessments of the cost burden of environmental health failures on local health systems would also deepen policy insights. Further research should examine how dynamics influence the ability of municipalities to enforce environmental health regulations in the presence of strong economic interests.

Although the study focuses on the Obuasi Municipality, several findings may have relevance beyond this specific setting. The authority-capacity mismatch observed in this study reflects structural characteristics commonly associated with decentralized governance systems in resource-dependent economies. Similarly, patterns of reactive community participation and enforcement challenges have been documented in other mining regions across sub-Saharan Africa. However, some aspects of the findings, particularly the specific institutional dynamics between municipal authorities and national regulators, may reflect contextual features unique to Ghana's decentralization framework. Similar governance patterns have been reported in other resource-dependent regions where decentralized institutions operate alongside powerful extractive industries. These parallels suggest that the authority-capacity mismatch observed in Obuasi may reflect broader structural features of decentralized governance in extraction-based economies rather than conditions unique to this municipality alone.

## Ethics statement

Ethical approval for the study was obtained from the University of Ghana School of Business with reference number: UGBS/COAB/20.05.07.

## Consent for publication

Not applicable.

## Availability of data and materials

The qualitative data generated and analyzed during the current study are not publicly available due to confidentiality and ethical restrictions, as they contain information that could potentially identify participants. Anonymized data excerpts may be made available from the corresponding author upon reasonable request.

## Funding

This research did not receive any specific grant from funding agencies in the public, commercial, or not-for-profit sectors.

## Declaration of competing interest

None.
